# Supplementary material for: Circadian Rhythm and Sleep Analyses in a Fruit Fly Model of Fragile X Syndrome Using a Video-Based Automated Behavioral Research System
Source: Int J Mol Sci. 2024 Jul 20;25(14):7949. doi: 10.3390/ijms25147949 (PMC11277495; doi:10.3390/ijms25147949)
Supplement: Supplementary file 1 [file ijms-25-07949-s001.zip › ijms-3055654-supplementary.pdf]

Supplementary table S1. Sleep analysis for dark-dark. Sleep parameters for female and male flies in w^{1118}, per^{01} *and* $dFMR1^{B55}$ groups, per day, in dark-dark (DD) period of experiments.

Sleep parameter	Sex	females				males			
	Phenotype	w^{1118}	*per01*	$dFMR1^{B55}$	p value	w^{1118}	*per01*	$dFMR1^{B55}$	p value
No of bouts	Mean ± SD	13.94 ± 10.61	8.78 ± 7.44	13.77 ± 7.7	**<0.0001**	17.55 ± 10.11	12.65 ± 7.78	19.08 ± 8.15	**<0.0001**
	Median [min-max]	13 [1 - 42]	6 [1 - 42]	13 [1 - 39]		17.5 [1 - 46]	12 [1 - 41]	20 [1 - 52]	
Sleep bout length (min)	Mean ± SD	10.07 ± 6.02	21.15 ± 21.43	24.03 ± 31.69	**<0.0001**	10.91 ± 5.02	23.02 ± 20.68	25.81 ± 33.03	**<0.0001**
	Median [min-max]	8.38 [0 - 65.5]	13.8 [5 - 211]	16.3 [5 - 348.5]		9.39 [5 - 41.5]	17.53 [5 - 253]	19.87 [5 - 546]	
TST (min)	Mean ± SD	120.1 ± 147.2	144.5 ± 163.3	263.7 ± 176	**<0.0001**	190.1 ± 153.6	238.3 ± 162.3	404.7 ± 189.4	**<0.0001**
	Median [min-max]	45 [0 - 572]	77 [0 - 691]	265 [0 - 713]		156 [0 - 621]	205.5 [0 - 692]	451.5 [0 - 713]	

Abbreviations: w^{1118} - *white*^{1118}; per^{01} - *period*^{01}; $dFMR1^{B55}$ -*dFMR1* mutant flies; min – minute; TST - total sleeping time; p value < or = 0.05 is considered statistically significant
